# *O-*Glycosylation Signatures Shape the Tumour Immune Microenvironment and Associate with Genomic Stability, Drug Resistance Programmes, and Epithelial Differentiation in Colorectal Cancer

**DOI:** 10.3390/ph19060857

**Published:** 2026-05-29

**Authors:** Abdullah A. Alqasem, Glowi Alasiri, Ayoub Al Othaim, Abdulhadi M. Abdulwahed, Ahmad A. Alghamdi, Abdulkarim S. Binshaya, Abdulaziz Alfahed

**Affiliations:** 1 Department of Medical Laboratory, College of Applied Medical Sciences, Prince Sattam Bin Abdulaziz University, Al Kharj 11942, Saudi Arabia; a.alqasem@psau.edu.sa (A.A.A.); abd.aldosari@psau.edu.sa (A.S.B.); 2 Department of Biochemistry, College of Medicine, Imam Mohammad Ibn Saud Islamic University (IMSIU), Riyadh 13317, Saudi Arabia; gaalasiri@imamu.edu.sa; 3 Department of Medical Laboratories, College of Applied Medical Sciences, Majmaah University, Al-Majmaah 11952, Saudi Arabia; ay.alothaim@mu.edu.sa; 4 Department of Clinical Laboratory Sciences, College of Applied Medical Sciences, King Saud University, Riyadh 12372, Saudi Arabia; aabdulwahed@ksu.edu.sa; 5 Department of Clinical Laboratory Sciences, College of Applied Medical Sciences, Taif University, Taif 21944, Saudi Arabia; a.ghamdi@tu.edu.sa

**Keywords:** tumour immune microenvironment, *O-*glycosylation, colorectal cancer, immune-desert phenotype, genomic instability, multidrug resistance, epithelial differentiation

## Abstract

**Background/Objectives**: The tumour immune microenvironment (TIME) critically influences colorectal cancer (CRC) progression and therapeutic response, yet mechanisms shaping immune phenotypes remain unclear. Mucin-type *O-*glycosylation regulates tumour–immune interactions at the cell surface. **Methods**: We analysed *O-*glycosylation activity in 988 colorectal cancer (CRC) tumours derived from three independent cohorts: The Cancer Genome Atlas (TCGA-CRC, n = 534), the Clinical Proteomic Tumour Analysis Consortium (CPTAC2-CRC, n = 106), and the Sidra–Leiden University Medical Center (Sidra-LUMC, n = 348). *O-*glycosylation activity was quantified using a transcriptomic gene signature and single-sample gene set enrichment analysis (ssGSEA). Tumours were stratified into high and low *O-*glycosylation groups based on the median score, and associations with immune phenotypes, genomic alterations, and tumour functional states were assessed. **Results**: High *O-*glycosylation tumours exhibited an immune-desert phenotype with reduced immune-inflamed (*p* = 3.65 × 10^−10^) and immune-excluded (*p* = 0.0070) signatures alongside increased immune-desert scores (*p* = 0.0049) and reduced Siglec signalling (*p* = 8.14 × 10^−5^). *O-*glycosylation was associated with genomic stability, including lower *TP53* mutation frequency (*p* = 0.0056), reduced aneuploidy (*p* = 0.0116), and decreased fraction of genome altered (*p* = 0.0309). High *O-*glycosylation tumours also showed upregulation of multidrug resistance programmes and reduced epithelial–mesenchymal transition (*p* = 0.0141) and proliferation (*p* = 0.0294). **Conclusions**: *O-*glycosylation defines a CRC subtype characterised by immune exclusion, genomic stability, and multidrug resistance, highlighting its potential as a biomarker and therapeutic target.

## 1. Introduction

The tumour immune microenvironment (TIME) represents a dynamic ecosystem that critically influences cancer progression, immune evasion, and therapeutic response in colorectal cancer (CRC) [1,2]. Composed of cellular elements—including cancer-associated fibroblasts, tumour-infiltrating lymphocytes, myeloid cells, and myeloid-derived suppressor cells—alongside non-cellular components such as extracellular matrix, abnormal vasculature, and signalling molecules, the TIME orchestrates complex interactions that can either promote or suppress anti-tumour immunity [3,4]. The immunosuppressive nature of the TIME, driven by mechanisms including immune checkpoint activation, metabolic reprogramming, and recruitment of suppressive cell populations, enables tumours to evade immune detection and represents a major barrier to effective immunotherapy [5,6]. Understanding the molecular determinants that shape distinct immune phenotypes—inflamed, excluded, and desert tumours—has therefore emerged as a priority for developing predictive biomarkers and therapeutic strategies to reprogramme the TIME toward immune activation [3,4,7].

While genomic alterations in driver genes such as *TP53*, *BRAF*, and mismatch repair components have been extensively characterised in CRC [8,9], less attention has been paid to post-translational modifications that regulate tumour–immune interactions at the cell surface [10,11]. Protein glycosylation represents one of the most abundant and functionally diverse post-translational modifications in eukaryotic cells, with mucin-type *O-*glycosylation playing a particularly prominent role in cancer due to its modification of cell surface proteins, including mucins, receptors, and adhesion molecules [10,11,12,13,14]. Aberrant *O-*glycosylation, frequently characterised by truncated glycan structures such as Tn and sialyl-Tn antigens, has been implicated in tumour progression, altered cell adhesion, and immune evasion [15,16,17,18,19,20].

In the context of the TIME, *O-*glycosylation serves as a critical mediator of cell–cell and cell–matrix interactions [21]. Glycan structures on tumour cells modulate immune recognition through multiple mechanisms. They influence antigen presentation, engage inhibitory lectin receptors such as Sialic acid-binding immunoglobulin-like lectins (Siglecs) expressed on immune cells, and form physical barriers that limit immune cell infiltration into the tumour parenchyma [20,22,23,24,25,26]. These glycosylation-dependent mechanisms contribute directly to the establishment of distinct immune phenotypes—inflamed, excluded, and desert tumours—which carry important prognostic and therapeutic implications [3]. Despite this mechanistic relevance, scalable approaches to quantify glycosylation states in large patient cohorts remain limited. Traditional glycomics methods are technically challenging and not amenable to population-scale datasets, creating a need for alternative strategies to infer glycosylation activity from widely available transcriptomic data [27].

In this study, we address this gap by performing a comprehensive systems-level analysis of *O-*glycosylation in CRC using transcriptomic data from 988 tumours with a particular focus on its role in shaping the TIME. Our specific objectives were to (i) quantify *O-*glycosylation activity using a single-sample gene set enrichment analysis (ssGSEA)-based gene expression signature; (ii) evaluate associations between *O-*glycosylation and clinicopathological features; (iii) assess relationships with key molecular alterations and genomic instability measures; (iv) determine the association between *O-*glycosylation and tumour immune phenotypes (inflamed, excluded, and desert); (v) investigate the relationship between *O-*glycosylation and multidrug resistance (MDR) programmes; and (vi) examine its association with tumour phenotypic states including proliferation, epithelial-mesenchymal transition (EMT), and epithelial differentiation. Through this integrated analysis, we sought to define the role of *O-*glycosylation as a potential intermediary linking tumour-intrinsic alterations to the regulation of the TIME in colorectal cancer.

## 2. Results

### 2.1. O-Glycosylation Activity Exhibits Substantial Heterogeneity in Colorectal Cancer

To quantify *O-*glycosylation activity in colorectal cancer, we applied single-sample gene set enrichment analysis using a curated gene signature comprising GALNT1, GALNT2, GALNT3, GALNT4, GALNT5, GALNT6, C1GALT1, and C1GALT1C1 (Supplementary Materials S1). *O-*glycosylation scores were successfully generated for 988 tumour samples, with 4 cases excluded due to missing expression data. The mean *O-*glycosylation score was 0.620 ± 0.139. The distribution exhibited substantial inter-tumour variability with a median of 0.629 (interquartile range, 0.581–0.665), consistent with marked heterogeneity in *O-*glycosylation programme activity across colorectal cancer samples. Sensitivity analyses using multiple *O-*glycosylation cut-offs (10th, 25th, 75th percentiles and median) demonstrated consistent patterns of association across clinicopathological variables, indicating that the observed relationships are not dependent on a single threshold (Supplementary Materials S2). The cohort was subsequently stratified into high and low *O-*glycosylation groups based on the median score (*O-*glycosyl-high, n = 494; *O-*glycosyl-low, n = 494), enabling comparative analyses across clinicopathological, molecular, and phenotypic variables.

### 2.2. O-Glycosylation Associates with Clinicopathological Features

**Figure 1:**
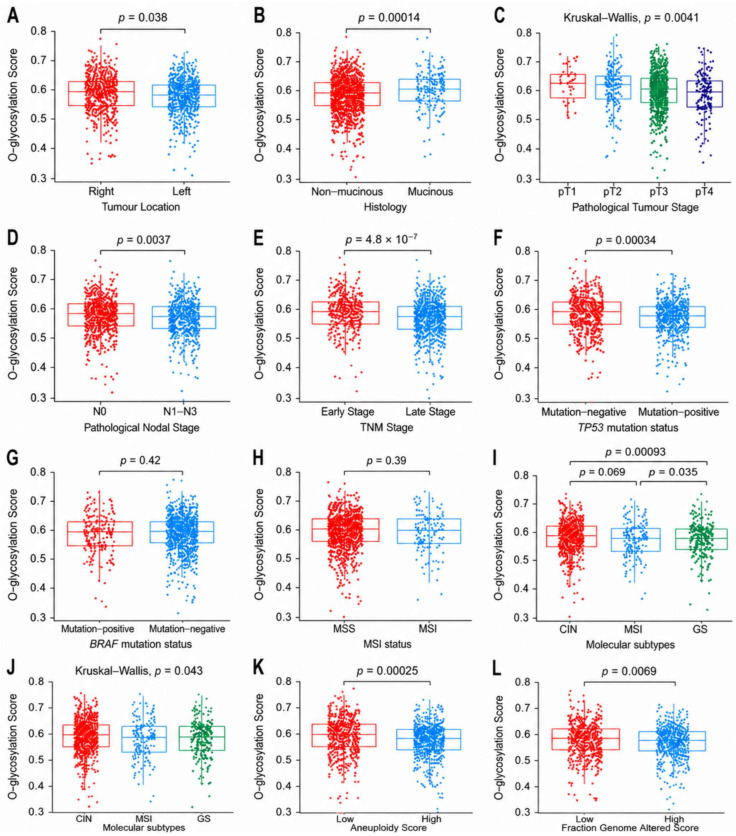
Clinicopathological and molecular/genomic correlates of O-glycosylation in colorectal cancer (CRC). Boxplots with jitter overlays showing higher O-glycosylation scores in (**A**) right-sided tumours, (**B**) mucinous tumours, (**C**) early pathological tumour stages, (**D**) early pathological nodal stages, and (**E**) early overall tumour–node–metastasis (TNM) stages. Higher O-glycosylation was also significantly associated with (**F**) *TP53* wild-type CRC, whereas no significant associations were observed with (**G**) *BRAF* mutation status or (**H**) microsatellite instability (MSI) status. In the molecular subtype analysis, O-glycosylation scores were relatively enriched in chromosomal instability (CIN)/epithelial-like tumours compared with MSI and genomically stable (GS) subtypes (**I,J**). Furthermore, higher O-glycosylation scores were associated with (**K**) low aneuploidy scores and (**L**) low fraction genome altered scores, indicating enrichment in CRC subsets with relatively lower genomic instability. Statistical comparisons were performed using the Mann–Whitney U test or Kruskal–Wallis test, and were confirmed using chi-square analysis where applicable. These findings suggest that O-glycosylation status defines biologically and genomically distinct CRC subsets. MSI, microsatellite instability; MSS, microsatellite stable; CIN, chromosomal instability; GS, genomically stable.

We evaluated the association between dichotomised *O-*glycosylation status and key clinicopathological parameters using chi-square tests (Figure 1, Supplementary Materials S3). *O-*glycosylation status showed a significant relationship with tumour location (Pearson χ^2^ = 3.847, *p* = 0.0498), with right-sided tumours demonstrating a higher proportion of *O-*glycosylation-high cases compared to left-sided tumours (53.2% vs. 46.9%). A stronger association was observed with histological subtype (Pearson χ^2^ = 13.590, *p* = 0.0002) as mucinous adenocarcinomas were markedly enriched for high *O-*glycosylation (63.6%), whereas non-mucinous tumours exhibited a lower prevalence (47.5%). *O-*glycosylation status also varied significantly across pathological T-stage categories (Pearson χ^2^ = 14.004, *p* = 0.0029), with early-stage tumours (pT1–pT2) more frequently classified as *O-*glycosylation-high compared to advanced tumours (pT3–pT4) (62.0% vs. 47.1%). This association remained consistent when the T-stage was dichotomised (Pearson χ^2^ = 13.679, *p* = 0.0002). Similarly, nodal status was significantly associated with *O-*glycosylation (Pearson χ^2^ = 4.111, *p* = 0.0426), with node-negative tumours showing a higher proportion of high *O-*glycosylation compared to node-positive disease (52.8% vs. 46.3%). At the level of overall disease stage, a strong association was evident (Pearson χ^2^ = 12.953, *p* = 0.0003), whereby early-stage tumours (Stage I–II) were more frequently *O-*glycosylation-high (57.5%), while late-stage tumours (Stage III–IV) were more commonly *O-*glycosylation-low (54.4%). In contrast, no significant association was observed between *O-*glycosylation status and metastatic stage (M0 vs. M1; Pearson χ^2^ = 0.730, *p* = 0.393). Furthermore, neither age at diagnosis (*p* = 0.146) nor gender (*p* = 0.373) demonstrated significant relationships with *O-*glycosylation status. Collectively, these findings indicate that elevated *O-*glycosylation is preferentially associated with right-sided, mucinous, and earlier-stage colorectal tumours, suggesting a link between *O-*glycosylation activity and less advanced disease phenotypes.

### 2.3. O-Glycosylation Relates to Molecular Alterations and Genomic Instability

We next assessed the relationship between *O-*glycosylation status and key molecular alterations as well as measures of genomic instability (Figure 1, Supplementary Materials S3). *O-*glycosylation demonstrated a significant association with *TP53* mutation status (Pearson χ^2^ = 7.664, *p* = 0.0056). Tumours with high *O-*glycosylation were more frequently *TP53* mutation-negative (51.5%), whereas low *O-*glycosylation tumours were enriched for *TP53* mutations (57.9%), indicating an inverse relationship between *O-*glycosylation activity and *TP53* mutational status. In contrast, no significant associations were observed between *O-*glycosylation and *BRAF* mutation status (Pearson χ^2^ = 0.310, *p* = 0.577) or microsatellite instability status (MSI) (Pearson χ^2^ = 0.810, *p* = 0.368). Similarly, *O-*glycosylation did not differ significantly across molecular subtypes (Pearson χ^2^ = 2.697, *p* = 0.260). Nonetheless, a modest trend was noted, with a higher proportion of *O-*glycosylation-high tumours within the epithelial/chromosomal instability (CIN) subtype (54.0%) compared to hypermutated/MSI (47.4%) and mesenchymal/EMT/genomically stable subtypes (48.4%). Notably, *O-*glycosylation was significantly associated with genomic instability metrics. High *O-*glycosylation tumours were more frequently observed in the low aneuploidy group compared to the high aneuploidy group (54.5% vs. 46.1%; Pearson χ^2^ = 6.370, *p* = 0.0116). A similar pattern was evident for fraction of genome altered (FGA), where high *O-*glycosylation was enriched in tumours with a low fraction of genome altered relative to those with high FGA (54.1% vs. 46.9%; Pearson χ^2^ = 4.660, *p* = 0.0309). Taken together, these findings indicate that elevated *O-*glycosylation is associated with a genomically more stable tumour phenotype characterised by lower *TP53* mutation frequency, reduced aneuploidy, and lower overall genomic disruption.

### 2.4. O-Glycosylation May Shape Tumour Immune Phenotypes

**Figure 2:**
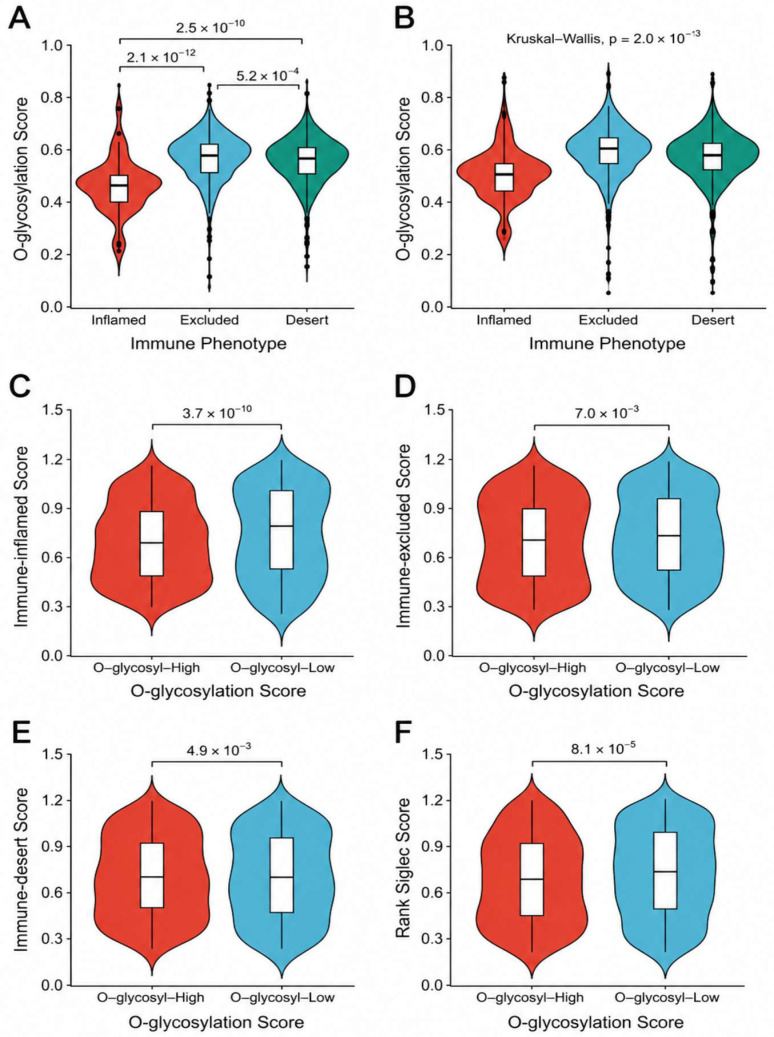
Association between the *O-*glycosylation score and tumour immune phenotypes. Violin plots with embedded boxplots showing the distribution of *O-*glycosylation scores across immune phenotypes and the relationship between the *O-*glycosylation stratification and immune-related signatures. (**A**,**B**) *O-*glycosylation scores are compared across three immune phenotypes (inflamed, excluded, and desert). *O-*glycosylation is significantly higher in excluded and desert tumours compared to inflamed tumours (pairwise *p*-values shown; Kruskal–Wallis test *p* = 2 × 10^−13^). (**C**,**D**) Tumours stratified into *O-*glycosylation-high and *O-*glycosylation-low groups show that the *O-*glycosylation-low group has significantly higher immune-inflamed and immune-excluded scores (*p* = 3.7 × 10^−10^ and *p* = 0.007, respectively). (**E**,**F**) *O-*glycosylation-high tumours are associated with significantly higher immune-desert scores (*p* = 0.0049) and lower Siglec scores (*p* = 8.1 × 10^−5^), whereas *O-*glycosylation-low tumours show the opposite pattern. Together, these results indicate that elevated *O-*glycosylation is associated with immune-excluded and immune-desert phenotypes, while lower *O-*glycosylation is linked to a more inflamed tumour microenvironment.

To characterise the TIME, we compared immune phenotype scores between high and low *O-*glycosylation groups using Mann–Whitney U tests (Table 1, Figure 2). High *O-*glycosylation tumours exhibited significantly lower immune-inflamed scores relative to low *O-*glycosylation tumours (Z, standardised test statistic = −6.268, *p* = 3.65 × 10^−10^), indicating reduced immune activation and cytotoxic activity within the TIME. A similar pattern was observed for the immune-excluded phenotype, which was also significantly diminished in the high *O-*glycosylation group (Z = −2.697, *p* = 0.0070). This reduction in immune-excluded scores suggests that high *O-*glycosylation tumours are not characterised by immune cells trapped at the tumour periphery but rather by a global reduction in immune presence. In contrast, immune-desert scores were significantly elevated in high *O-*glycosylation tumours (Z = 2.816, *p* = 0.0049), suggesting a relative absence of immune cell infiltration throughout the tumour parenchyma. This pattern—low inflamed, low excluded, and high desert—defines a canonical immune-desert phenotype, wherein tumours lack significant immune engagement at any level. Correlative analysis of immune phenotypes revealed a clear gradient relationship with *O-*glycosylation activity. *O-*glycosylation scores were negatively correlated with the immune-inflamed phenotype (r, Pearson’s rank correlation coefficient = −0.262, *p* < 0.001) and, to a lesser extent, the immune-excluded phenotype (r = −0.092, *p* = 0.004) while showing a positive correlation with the immune-desert phenotype (r = 0.122, *p* < 0.001). These findings indicate that increasing *O-*glycosylation activity is associated with reduced immune engagement and a shift towards an immune-desert tumour microenvironment.

### 2.5. Siglec-Related Signalling: Composite Score Associations and Gene-Level Correlation Structure

Siglec-related immune scores were significantly lower in high *O-*glycosylation tumours (Z = −3.940, *p* = 8.14 × 10^−5^), indicating reduced engagement of Siglec-mediated immune regulatory pathways. Collectively, these findings demonstrate that elevated *O-*glycosylation is associated with a predominantly immune-desert tumour phenotype characterised by diminished inflammatory and immune-excluded signatures alongside reduced activation of Siglec-associated immune modulation pathways. This suggests that *O-*glycosylation programmes are associated with an immunologically “cold” TIME in colorectal cancer. Given the established role of sialylated glycans as ligands for Siglec receptors, we further interrogated the relationship between *O-*glycosylation activity and Siglec-related signalling. Initial analyses demonstrated a significant inverse correlation between *O-*glycosylation and the composite Siglec score (r = −0.121, *p* < 0.001). This relationship remained unchanged following partial correlation analysis controlling for immune phenotype (r = −0.146, *p* < 0.001), indicating that the association was not fully attributable to differences in immune contexture. To explore whether aggregation of multiple Siglec genes into a composite score might obscure receptor-specific relationships, we examined the expression patterns of individual Siglec-related genes. Pairwise correlation analysis revealed strong positive co-expression among *CD33, SIGLEC7, SIGLEC9*, and *SIGLEC10* (r = 0.63–0.90, all *p* < 0.001), indicating that the composite Siglec score reflects a coordinated transcriptional programme. Subsequently, we assessed the relationship between *O-*glycosylation and each individual Siglec gene. *O-*glycosylation activity showed significant inverse correlations with *CD33* (r = −0.128, *p* < 0.001), *SIGLEC7* (r = −0.118, *p* < 0.001), and *SIGLEC9* (r = −0.198, *p* < 0.001), while no significant association was observed with *SIGLEC10* (r = −0.035, *p* = 0.267). These findings indicate that the inverse relationship between *O-*glycosylation and Siglec signalling is preserved at the individual gene level and is not an artefact of composite score construction. In summary, high *O-*glycosylation tumours exhibited lower Siglec-related scores, consistent with their enrichment for immune-desert phenotypes in CRC. Notably, Siglec-related scores and the expression levels of *CD33, SIGLEC7, SIGLEC9*, and *SIGLEC10* were significantly higher in immune-desert tumours compared with immune-inflamed tumours. To further interrogate this unexpected relationship, we examined the association between Siglec signalling and an independent sialylation score. In contrast to *O-*glycosylation, the sialylation score demonstrated a positive correlation with the Siglec score (r = 0.269, *p* < 0.001) and was positively associated with immune-inflamed and immune-excluded phenotypes, while showing a negative association with the immune-desert phenotype. Notably, no significant correlation was observed between *O-*glycosylation and sialylation scores. Collectively, these findings indicate that distinct glycosylation-related transcriptional programmes exhibit divergent relationships with Siglec signalling. While *O-*glycosylation is inversely associated with Siglec-related scores and enriched in immune-desert tumours, sialylation demonstrates concordant positive associations with both Siglec signalling and immune-active phenotypes.

### 2.6. O-Glycosylation Is Associated with Multidrug Resistance Programmes

**Figure 3:**
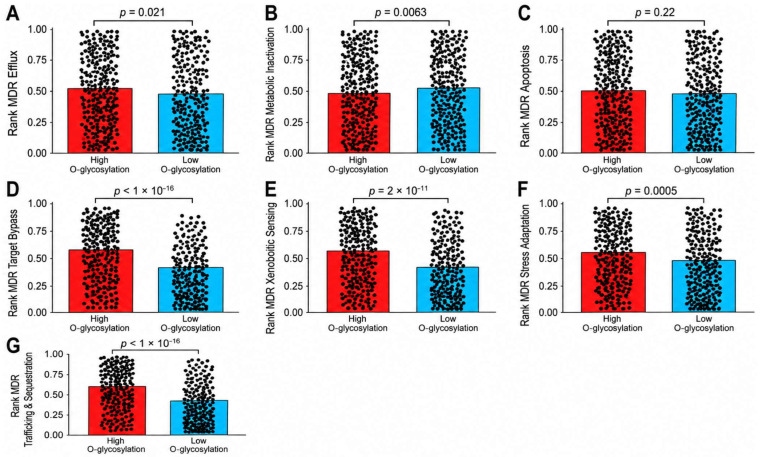
Association between *O-*glycosylation status and MDR programme signatures. Box-and-jitter plots showing the distribution of fractional-ranked (0–1 scale) MDR-related signature scores across *O-*glycosylation-high and *O-*glycosylation-low groups. Each dot represents an individual case, while boxes indicate the interquartile range with median values. Compared to the low group, *O-*glycosylation-high tumours demonstrate significantly higher scores for multiple MDR programmes including (**A**) MDR efflux (*p* = 0.021), (**D**) target bypass (*p* < 2.22 × 10^−16^), (**E**) xenobiotic sensing (*p* = 2 × 10^−11^), (**F**) stress adaptation (*p* = 0.0005), and (**G**) drug trafficking and sequestration (*p* < 2.22 × 10^−16^), while low *O*-glycosylation tumours exhibit higher scores for (**B**) metabolic inactivation (*p* = 0.0063). No significant difference is seen for (**C**) MDR-associated apoptosis (*p* = 0.22). Statistical comparisons were performed using the Mann–Whitney U test. These results indicate that elevated *O-*glycosylation is broadly associated with enrichment of multiple MDR mechanisms. MDR = multiple drug resistance.

We systematically evaluated the relationship between *O-*glycosylation status and seven MDR programmes using Mann–Whitney U tests (Figure 3, Table 2). High *O-*glycosylation tumours demonstrated a broad enrichment of several key MDR mechanisms. Specifically, MDR efflux activity reflecting ATP-binding cassette (ABC) transporter function was significantly increased in the high *O-*glycosylation group (Z = 2.311, *p* = 0.0208) alongside a marked upregulation of target bypass signalling (Z = 8.287, *p* < 0.0001), indicating enhanced capacity for tumours to circumvent therapy through alternative pathway activation. Similarly, stress adaptation pathways were significantly elevated (Z = 3.479, *p* = 0.0005), as were xenobiotic sensing mechanisms (Z = 6.707, *p* = 1.99 × 10^−11^) and drug trafficking/sequestration processes (Z = 9.485, *p* < 0.0001). These findings indicate that high *O-*glycosylation tumours possess enhanced capabilities for detecting and responding to xenobiotic stress, as well as sequestering chemotherapeutic agents away from their intracellular targets. In contrast, metabolic inactivation pathways—representing phase I/II drug metabolism enzymes—were significantly reduced in high *O-*glycosylation tumours (Z = −2.730, *p* = 0.0063), suggesting a selective rather than uniform activation of MDR programmes. No significant difference was observed for apoptosis suppression between high and low *O-*glycosylation groups (*p* = 0.2189). Collectively, these findings indicate that elevated *O-*glycosylation is associated with a coordinated upregulation of multiple MDR mechanisms, particularly those related to drug transport, adaptive stress responses, and signalling plasticity, while sparing apoptosis-related resistance pathways. This pattern supports a model in which *O-*glycosylation is associated with a multifaceted, non-canonical drug resistance phenotype in colorectal cancer, with potential implications for therapeutic response and treatment stratification. Bivariate correlation analyses confirm these associations. *O-*glycosylation activity was positively associated with multiple MDR programmes. Strong correlations were observed with trafficking/sequestration (r = 0.402, *p* < 0.001) and target bypass signalling (r = 0.365, *p* < 0.001) alongside moderate associations with stress adaptation (r = 0.232, *p* < 0.001) and xenobiotic sensing (r = 0.217, *p* < 0.001). Weaker but statistically significant correlations were also observed with apoptosis suppression (r = 0.139, *p* < 0.001) and efflux mechanisms (r = 0.092, *p* = 0.004). In contrast, no significant association was observed with metabolic inactivation (r = −0.03, *p* = 0.346). These findings indicate that *O-*glycosylation is associated with a broad but non-uniform MDR phenotype, characterised by stronger engagement of signalling, trafficking, and adaptive resistance pathways, with limited involvement of drug metabolism processes.

### 2.7. O-Glycosylation Is Associated with Tumour Phenotypic States

**Figure 4:**
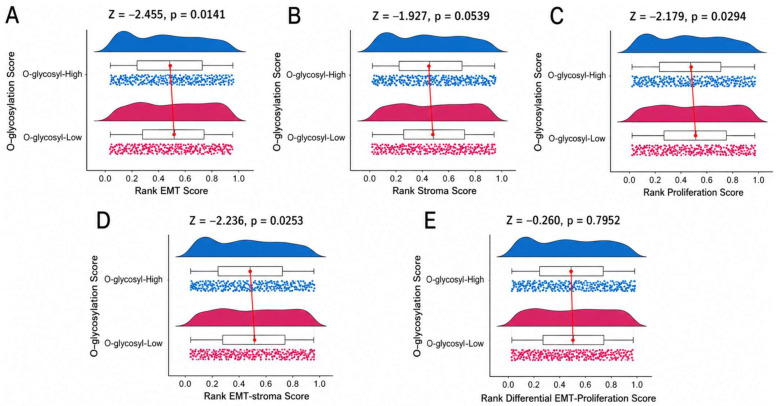
Comparison of ranked tumour microenvironment scores between *O-*glycosylation subgroups. Raincloud plots illustrating the distribution of fractional-ranked scores (0–1 scale) for EMT, stroma, proliferation, EMT–stroma composite, and differential EMT–proliferation signatures in *O-*glycosylation-high and *O-*glycosylation-low groups. Each panel shows the density distribution (top), boxplot (centre), and individual data points (bottom), with red lines indicating paired differences in central tendency between groups. Across signatures, the *O-*glycosylation-high group demonstrates a consistent shift toward lower (**A**) EMT (Z = −2.455, *p* = 0.0141), (**C**) proliferation (Z = −2.179, *p* = 0.0294), and (**D**) EMT–stroma composite scores (Z = −2.236, *p* = 0.0253), with a borderline increase in (**B**) stromal scores (Z = −1.927, *p* = 0.0539). No significant difference was observed for (**E**) the differential EMT–proliferation score (Z = −0.260, *p* = 0.7952). Statistical comparisons were performed using the Mann–Whitney U test. These findings indicate that low *O-*glycosylation is associated with enrichment of EMT-related and proliferative tumour phenotypes. Negative Z-statistics denote lower values in the *O-*glycosylation-high group compared with the *O-*glycosylation-low group. Distributions and boxplots reflect this directionality.

**Figure 5:**
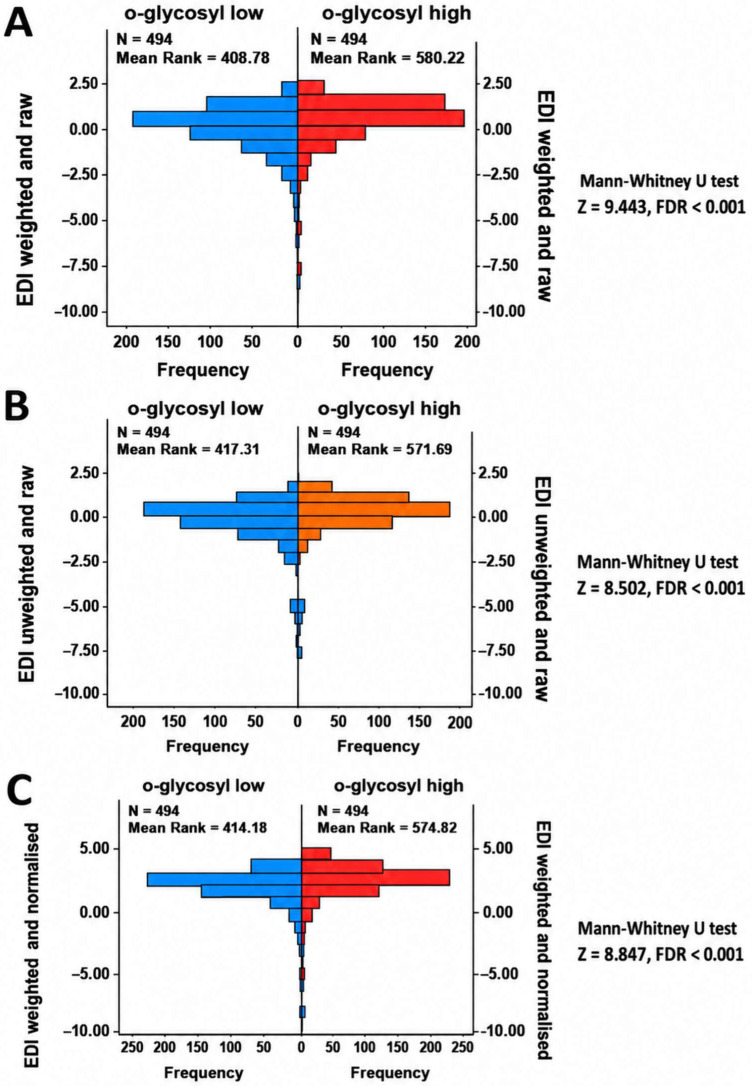
Association between *O-*glycosylation status and EDI scores. Mirror histograms showing the distribution of EDI scores across *O-*glycosylation-low and *O-*glycosylation-high groups (N = 494 per group). (**A**) weighted and raw EDI scores, (**B**) unweighted and raw EDI scores, (**C**) weighted and normalised EDI scores. In all comparisons, cases in the *O-*glycosylation-high group exhibit a rightward shift, indicating higher EDI values relative to the low group, consistent with higher mean ranks. Differences between groups were assessed using the Mann–Whitney U test, demonstrating statistically significant differences for both raw (Z, standardised test statistic = 9.443, false discovery rate (FDR) < 0.001) and normalised scores (Z = 8.847, FDR < 0.001). These findings indicate a strong association between elevated *O-*glycosylation and EDI scores. EDI = epithelial differentiation index.

We next examined the relationship between *O-*glycosylation and key tumour phenotypic states, including proliferation, EMT, and differentiation (Figure 4, Supplementary Materials S4). In all comparisons, negative Z-values indicate lower scores in the *O-*glycosylation-high group relative to the *O-*glycosylation-low group. High *O-*glycosylation tumours exhibited significantly lower EMT scores compared to their low *O-*glycosylation counterparts (Z = −2.455, *p* = 0.0141), indicating reduced mesenchymal transition and maintenance of epithelial characteristics. In parallel, proliferation scores were also significantly decreased in the high *O-*glycosylation group (Z = −2.179, *p* = 0.0294), suggesting a less proliferative, more quiescent tumour phenotype. Stromal content showed a similar downward trend in high *O-*glycosylation tumours. However, this did not reach statistical significance (Z = −1.927, *p* = 0.0539). Notably, the composite EMT–stroma score was significantly lower in the high *O-*glycosylation group (Z = −2.236, *p* = 0.0253), reinforcing the observation of a less mesenchymal and less stromally enriched tumour state. This pattern suggests that high *O-*glycosylation tumours maintain a compact epithelial architecture with limited desmoplastic reaction. In striking contrast, epithelial differentiation was markedly enhanced in high *O-*glycosylation tumours. All three measures of the epithelial differentiation index (EDI) demonstrated strong and consistent elevation (Z = 9.443, 8.502, and 8.847, respectively; all *p* < 0.000, Figure 5), representing one of the most robust associations observed in the analysis. This finding indicates that high *O-*glycosylation is tightly linked to well-differentiated tumour histology, consistent with the earlier observation of enrichment in mucinous tumours, which retain glandular differentiation. No significant difference was identified in the EMT–proliferation differential score between groups (*p* = 0.7952), suggesting that the balance between these two programmes remains stable despite individual reductions. Taken together, these findings indicate that elevated *O-*glycosylation is strongly associated with a well-differentiated, epithelial tumour phenotype characterised by reduced EMT, diminished proliferative activity, and pronounced epithelial differentiation, with minimal impact on the balance between EMT and proliferation programmes. This phenotypic profile aligns with the immune-desert TIME and suggests that *O-*glycosylation is associated with both tumour cell-intrinsic properties and microenvironmental interactions.

Correlation analysis demonstrated that *O-*glycosylation activity is associated with epithelial differentiation and tumour phenotypic programmes. *O-*glycosylation scores showed consistent positive correlations with epithelial differentiation indices, including weighted and raw EDI scores (r = 0.319, *p* < 0.001), unweighted and raw EDI scores (r = 0.293, *p* < 0.001), and weighted and normalised EDI scores (r = 0.296, *p* < 0.001). In parallel, *O-*glycosylation was inversely associated with mesenchymal and stromal transcriptional programmes. Significant negative correlations were observed with epithelial–mesenchymal transition (EMT) (r = −0.130, *p* < 0.001), stromal scores (r = −0.085, *p* = 0.008), and EMT–stroma composite scores (r = −0.110, *p* < 0.001). In contrast, no significant association was observed with proliferation scores (r = −0.033, *p* = 0.304). Together, these findings indicate that *O-*glycosylation activity is positively associated with epithelial differentiation and inversely associated with EMT and stromal features, while showing no relationship with tumour proliferative activity.

### 2.8. Association of O-Glycosylation with Survival

Exploratory survival analyses were performed using both median and 75th percentile cut-offs of the *O-*glycosylation score. No statistically significant differences in overall survival were observed. However, a trend toward reduced survival was noted in the high *O-*glycosylation group using the median cut-off (log-rank χ^2^ = 3.309, *p* = 0.069) while the 75th percentile cut-off showed no significant association (log-rank χ^2^ = 2.706, *p* = 0.100). In univariate Cox analysis, *O-*glycosylation was significantly associated with survival (B, regression coefficient = −2.186, *p* = 0.008; HR, hazard ratio = 0.112). However, in multivariable Cox regression including overall tumour stage, *O-*glycosylation was not independently associated with survival (B = −1.473, *p* = 0.082; HR = 0.229), whereas overall stage remained a strong predictor (B = 0.675, *p* < 0.001; HR = 1.964).

### 2.9. Integrated Summary of O-Glycosylation-Associated Tumour Architecture in Colorectal Cancer

**Figure 6:**
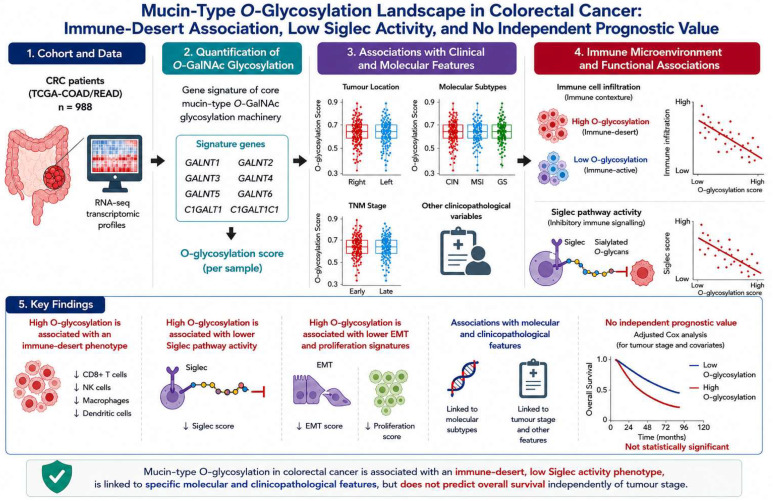
Transcriptomic profiling of colorectal cancer (CRC) tumours (n = 988) was used to quantify mucin-type *O*-glycosylation activity using a curated gene signature (GALNT1–6, C1GALT1, and C1GALT1C1) and ssGSEA. Tumours were stratified into high and low *O*-glycosylation groups, revealing a distinct biological phenotype. High *O*-glycosylation tumours were characterised by an immune-desert tumour microenvironment, with reduced immune-inflamed and immune-excluded signatures and increased immune-desert features. These tumours also exhibited genomic stability, including lower *TP53* mutation frequency, reduced aneuploidy, and decreased FGA. Functionally, high *O-*glycosylation was associated with a well-differentiated epithelial state, marked by reduced EMT and proliferation, alongside enhanced epithelial differentiation. Concurrently, multiple MDR programmes were upregulated, including drug efflux, target bypass signalling, stress adaptation, xenobiotic sensing, and drug trafficking/sequestration. Collectively, *O*-glycosylation defines a colorectal cancer subtype characterised by immune exclusion, genomic stability, and therapeutic resistance potential, highlighting its relevance as a biomarker and candidate target for precision oncology. TNM, tumour, node and metastasis.

Across 988 colorectal cancers, high *O-*glycosylation defined a coherent tumour phenotype spanning clinicopathological, molecular, immune, and functional dimensions (Supplementary Materials S5). High *O-*glycosylation tumours were enriched in right-sided, mucinous, and earlier-stage disease and exhibited features of genomic stability, including lower *TP53* mutation frequency, reduced aneuploidy, and decreased FGA. At the phenotypic level, *O-*glycosylation was strongly associated with epithelial differentiation, with reduced EMT and stromal programmes and no significant association with proliferation. In the TIME, high *O-*glycosylation was linked to an immune-desert phenotype, with reduced immune-inflamed and immune-excluded signatures and lower Siglec-related transcriptional scores. In contrast, sialylation correlated positively with immune-active phenotypes and Siglec signalling, indicating divergence between glycosylation axes. Functionally, high *O-*glycosylation tumours demonstrated coordinated enrichment of multiple MDR programmes. Although associated with survival in univariate analysis, *O-*glycosylation was not an independent prognostic factor after adjustment for tumour stage. These integrated relationships are summarised in Figure 6.

## 3. Discussion

The TIME represents a critical determinant of cancer progression and therapeutic response, yet the molecular mechanisms that establish and maintain distinct immune phenotypes remain incompletely characterised [28,29]. We demonstrate that *O-*glycosylation signatures are strongly associated with TIME architecture in colorectal cancer, identifying elevated *O-*glycosylation activity as a feature of immune-desert tumours characterised by reduced inflammatory infiltrates, diminished immune-excluded patterns, and global immune absence. These findings extend our understanding of how tumour-intrinsic glycosylation programmes shape immune recognition and exclusion, with important implications for immunotherapy stratification and combination strategies [17,19,20,30,31,32].

The association between high *O-*glycosylation and immune-desert phenotype is particularly noteworthy given the mechanistic links between cell surface glycans and immune cell recognition [22]. Glycan structures, particularly truncated *O-*glycans such as Tn and sialyl-Tn antigens, have been shown to modulate immune responses through multiple mechanisms. They can mask underlying peptide epitopes from antigen recognition, engage inhibitory lectin receptors, including Siglecs on immune cells, and contribute to the formation of physical barriers such as the glycocalyx that impede immune cell penetration [17,19,33,34]. Our observation of reduced Siglec pathway engagement in high *O-*glycosylation tumours may initially seem counterintuitive, as sialylated glycans typically serve as Siglec ligands [24,25,26]. However, this finding may reflect qualitative differences in glycan structures—such as the prevalence of truncated versus elongated *O-*glycans—or context-dependent effects on immune receptor engagement that warrant further investigation [35,36]. On the other hand, the inverse association between O-glycosylation activity and Siglec-related scores can be interpreted within the context of the immune-desert phenotype observed in high O-glycosylation tumours (Figure 2). Siglec-related genes, including *CD33*, *SIGLEC7*, *SIGLEC9*, and *SIGLEC10*, are predominantly expressed by immune cell populations such as myeloid cells and natural killer (NK) cells [22,24,25,26]. Consequently, their expression in bulk transcriptomic data is strongly influenced by the abundance and composition of infiltrating immune cells within the tumour microenvironment [22,24,25,26]. The strong co-expression observed among these genes (r ≈ 0.63–0.90) further supports the interpretation that the composite Siglec score reflects a coordinated immune cell–associated transcriptional signal rather than tumour-cell intrinsic glycosylation alone. Accordingly, the reduced Siglec-related scores observed in high O-glycosylation tumours are consistent with diminished immune infiltration characteristic of an immune-desert tumour microenvironment rather than reduced glycan–Siglec interactions per se. Importantly, elevated mucin-type O-glycosylation does not necessarily imply increased terminal glycan sialylation [10,11,24,25]. The lack of correlation observed between O-glycosylation and sialylation scores suggests that the O-glycosylation programme captured in this study is not enriched for terminally sialylated glycans. This distinction is biologically relevant because Siglec receptors specifically recognise sialylated glycan structures [22,24,25,26]. Therefore, the inverse association between O-glycosylation and Siglec-related scores may partly reflect the separation between early O-glycan biosynthetic scaffolding pathways and terminal sialylation processes. The composition of the O-glycosylation signature used in this study further supports this interpretation. The score comprises N-acetylgalactosaminyltransferase (GALNT) family members (GALNT1–6), which initiate mucin-type O-glycosylation, together with C1GALT1/C1GALT1C1, which mediate core 1 glycan formation [10,11]. Notably, this signature captures early stages of O-glycan biosynthesis and does not include enzymes directly responsible for terminal glycan sialylation [10,11,24,25]. Thus, the O-glycosylation programme quantified here likely reflects glycan scaffold generation rather than terminally sialylated glycan structures. This distinction provides a plausible biological explanation for the inverse association observed between O-glycosylation and Siglec-related scores, despite the positive association observed with the sialylation score. Nevertheless, the persistence of this inverse association after adjustment for immune phenotype suggests that it is not fully explained by categorical immune classifications alone and may additionally reflect more subtle variations in immune cell density or composition. Furthermore, as the present analysis is based on transcriptional proxies rather than direct glycomic profiling, the relative contribution of sialylated versus non-sialylated O-glycan structures cannot be definitively determined and warrants further investigation.

The relationship between *O-*glycosylation and genomic stability observed in our study—characterised by lower *TP53* mutation frequency, reduced aneuploidy, and decreased FGA—suggests that *O-*glycosylation programmes may be preferentially maintained in tumours with less genomic chaos. This aligns with the association between high *O-*glycosylation and well-differentiated, epithelial phenotypes exhibiting reduced EMT and proliferation [13,14]. One interpretation is that *O-*glycosylation represents a feature of “differentiation-addicted” tumours that retain aspects of normal epithelial programming, rather than those that have undergone dedifferentiation through genomic instability and EMT [37,38]. The enrichment of high *O-*glycosylation in mucinous tumours, which by definition maintain glandular differentiation and mucin production, further supports this conceptual framework [39].

The coordinated upregulation of MDR programmes in high *O-*glycosylation tumours carries potential therapeutic implications. The selective activation of efflux activity, target bypass signalling, stress adaptation, xenobiotic sensing, and drug trafficking—while sparing metabolic inactivation and apoptosis suppression—suggests a specific MDR configuration that may influence response to conventional chemotherapy and targeted agents [40,41]. These findings are consistent with established mechanisms of MDR mediated by ABC transporters, which actively efflux chemotherapeutic agents and reduce intracellular drug accumulation [42,43,44]. The mechanistic connections between *O-*glycosylation and these resistance programmes remain to be fully elucidated but may involve glycosylation-dependent regulation of transporter stability, signalling receptor function, or stress response pathways [45]. From a clinical perspective, these findings raise the possibility that *O-*glycosylation status could inform treatment stratification, with high *O-*glycosylation tumours potentially requiring alternative therapeutic approaches to overcome their intrinsic resistance programmes [18,20,46,47,48].

The association between *O-*glycosylation and clinicopathological features—right-sided location, mucinous histology, earlier stage, and node-negative status—paints a coherent picture of a distinct tumour subtype. Right-sided CRCs have been associated with different molecular features, including higher rates of MSI, *BRAF* mutation, and CpG island methylator phenotype; yet our analysis did not find significant associations with these features [49,50]. This suggests that *O-*glycosylation may define an orthogonal axis of biological variation that cuts across existing molecular classifications, potentially capturing aspects of tumour differentiation and microenvironmental interaction not fully captured by genomic features alone [51,52]. Although univariate analysis suggested an association between *O-*glycosylation and survival, this effect was not retained after adjustment for tumour stage in multivariable modelling. These findings indicate that *O-*glycosylation is not an independent prognostic factor, and that its association with survival is likely mediated through its relationship with established clinicopathological features such as tumour stage.

Several limitations of this study should be acknowledged. First, our *O-*glycosylation signature is based on transcript abundance of glycosylation enzymes, which may not fully capture the complexity of glycan structures resulting from post-transcriptional regulation, enzyme competition, and substrate availability [53]. Second, the use of bulk transcriptomic data precludes dissection of cell type-specific glycosylation patterns within the tumour ecosystem, an important consideration given that glycosylation programmes differ between epithelial tumour cells, stromal fibroblasts, and immune populations. Accordingly, the glycosylation-related scores reported in this study represent aggregate transcriptional signals across multiple cellular compartments rather than cell-intrinsic activity. Future studies employing single-cell or spatially resolved approaches will be required to disentangle these contributions [54]. Third, while our modular approach to immune phenotyping captures established signatures, validation with orthogonal methods such as multiplex immunohistochemistry or spatial transcriptomics would strengthen the association between *O-*glycosylation and immune-desert architecture [55,56]. Fourth, the observational nature of this study limits causal inference; functional studies using in vitro and in vivo models are needed to establish whether *O-*glycosylation actively drives immune exclusion or represents a passenger phenomenon in well-differentiated tumours [57,58].

Despite these limitations, our findings have several implications for future research and clinical translation. The identification of *O-*glycosylation as a robust correlate of immune-desert phenotype suggests that glycosylation signatures could serve as biomarkers for immunotherapy response prediction, complementing existing markers such as MSI status, tumour mutational burden, and Programmed Death-Ligand 1 (PD-L1) expression [59,60]. Patients with high *O-*glycosylation tumours, predicted to have low baseline immune infiltration, may be less likely to respond to immune checkpoint inhibitors as monotherapy and could be prioritised for combination strategies aimed at remodelling the TIME, such as glycosylation-modifying agents, oncolytic viruses, or therapies targeting myeloid suppression [61,62]. Additionally, the association with MDR programmes suggests that *O-*glycosylation status could inform chemotherapy selection and the use of resistance-modifying agents [42,43].

## 4. Materials and Methods

### 4.1. Data Acquisition and Processing

Transcriptomic and corresponding clinical data for colorectal cancer were obtained from three independent public cohorts, including The Cancer Genome Atlas (TCGA-CRC, n = 534), the Clinical Proteomic Tumour Analysis Consortium (CPTAC2-CRC, n = 110), and the Sidra–Leiden University Medical Center (Sidra-LUMC, n = 348) [8,46,47]. Only samples from primary colorectal adenocarcinomas with complete transcriptomic (n = 988) and clinical annotations were considered for inclusion. To create a unified, harmonised dataset for analysis, gene identifiers were standardised across all platforms, and genes not consistently present in all three cohorts were excluded, resulting in a final set of 11,731 genes for downstream analysis. Potential batch effects arising from technical differences between the cohorts were corrected using the ComBat algorithm from the sva R package (version 4.5.2) [63]. The effectiveness of this correction was visually confirmed through principal component analysis, which showed a loss of cohort-specific clustering post-correction (Supplementary Figure S1).

### 4.2. Quantification of O-Glycosylation Activity

To quantify *O-*glycosylation activity, we curated a gene signature representing the core machinery of mucin-type *O-*GalNAc glycosylation [10,11]. The final signature comprised genes encoding polypeptide *N*-acetylgalactosaminyltransferases (GALNT1-6), the core 1 synthase (C1GALT1), and its specific chaperone (C1GALT1C1). For each tumour sample, a continuous *O-*glycosylation enrichment score was generated from the normalised, harmonised expression data using ssGSEA, implemented via the GSVA R package [27,64]. This approach provides a robust, sample-specific measure of pathway activity. Descriptive statistics were calculated to characterise the distribution of this score across the entire study population. The cohort was subsequently stratified into high and low *O-*glycosylation groups based on the median score (*O-*glycosyl-high, n = 494; *O-*glycosyl-low, n = 494) to enable comparative analyses across clinicopathological, molecular, and phenotypic variables.

### 4.3. Construction of Tumour Microenvironment and Functional Modules

To investigate the biological context of *O-*glycosylation, we constructed a series of transcriptional modules representing key phenotypic and functional states relevant to the TIME. These modules, which served as proxies for the tumour microenvironment and cellular processes, were scored using either the average of standardised gene expression values or the ssGSEA method.

#### 4.3.1. Immune Phenotype Modules

To classify the TIME, we constructed modules for inflamed, excluded, and desert phenotypes based on established transcriptomic criteria [3]. The inflamed phenotype was characterised by signatures of cytotoxic immune activity, T-cell infiltration, and interferon-γ response; the excluded phenotype by stromal barriers and chemokine gradients limiting immune penetration; and the desert phenotype by global immune cell absence. Additionally, a Siglec-related immune score was generated to assess engagement of sialic acid-binding immunoglobulin-type lectin pathways mediating immune inhibition.

#### 4.3.2. Tumour State Modules

We built modules to assess proliferation (cell cycle and Ki-67-associated genes), EMT (core signature), stromal content (cancer-associated fibroblast and extracellular matrix genes), and epithelial differentiation (three complementary indices measuring glandular differentiation and epithelial integrity). Composite scores including EMT-stroma and EMT-proliferation differential were also calculated.

#### 4.3.3. Therapeutic Resistance Modules

Modules capturing mechanisms of MDR were constructed, including those related to drug efflux (ABC transporter family), metabolic inactivation (phase I/II drug metabolism enzymes), apoptosis evasion (anti-apoptotic BCL-2 family and inhibitors of apoptosis proteins), target bypass signalling (alternative pathway activation), stress adaptation (oxidative and xenobiotic stress responses), xenobiotic sensing (nuclear receptors and transcription factors), and drug trafficking/sequestration (vesicular transport and lysosomal sequestration) [33,34,35,41,65,66].

The complete list of genes comprising these modules is provided in Supplementary Materials S1. The transcriptional modules used in this study comprise a combination of previously validated gene signatures (immune phenotypes, EMT, proliferation, and stromal programmes) and custom-curated gene sets (*O-*glycosylation and MDR pathways), the latter constructed based on well-established biological mechanisms described in the literature.

### 4.4. Association with Clinicopathological and Molecular Features

To address our primary objectives, we systematically evaluated the relationship between *O-*glycosylation status and a wide range of variables. This included (i) clinicopathological features including pathological tumour (T), nodal (N), and metastasis (M) stages, histological subtype (mucinous vs. non-mucinous), tumour location (right-sided vs. left-sided), age at diagnosis, and gender; (ii) molecular alterations including mutation status of key driver genes (*TP53, BRAF*), MSI status, and established molecular subtype classifications (epithelial/CIN, hypermutated/MSI, mesenchymal/EMT/genomically stable); (iii) genomic instability measures of chromosomal instability including aneuploidy status (high vs. low) and FGA (high vs. low based on median cut-off). Variation in sample size across clinicopathological variables reflects missing data in specific annotations across the merged cohorts. Analyses were therefore performed using pairwise exclusion, whereby cases were included only if relevant data for the variable of interest were available.

### 4.5. Statistical Methodology

All statistical analyses were performed using IBM SPSS Statistics (version 29). Given the non-normal distribution of the *O-*glycosylation scores, as determined by Shapiro–Wilk testing, non-parametric methods were employed throughout. To assess the robustness of dichotomisation and potential threshold effects, sensitivity analyses were performed using alternative *O-*glycosylation cut-offs defined at the 10th, 25th, and 75th percentiles, in addition to the median (Supplementary Materials S2). For comparisons of continuous module scores (immune phenotypes, MDR programmes, and tumour states) between high and low *O-*glycosylation groups, the Mann–Whitney U test was used, with exact Z-scores and *p*-values reported. For categorical variables (clinicopathological features, mutation status, and genomic instability measures), associations with dichotomised *O-*glycosylation status were assessed using the chi-square test (Pearson χ^2^) or Fisher’s exact test where appropriate. For comparisons involving three or more groups (e.g., molecular subtypes and T-stage categories), the Kruskal–Wallis test was applied. Where the Kruskal–Wallis test was significant, post-hoc pairwise comparisons were conducted using Dunn’s test, with *p*-values adjusted for multiple testing using the Bonferroni correction. Spearman’s rank correlation coefficient was used to evaluate monotonic relationships between continuous variables such as *O-*glycosylation scores and other module scores. A two-sided *p*-value < 0.05 was considered statistically significant. For all post-hoc and multiple comparisons, adjusted *p*-values are reported as indicated.

### 4.6. Data Visualisation

Graphical representations were generated using SPSS and the SRPlot online platform [67]. Boxplots overlaid with individual data points (jitter) were used to visualise the distribution of module scores across *O-*glycosylation groups, displaying the median and interquartile range. Statistical significance from group comparisons was annotated directly on these figures. Enrichment analyses and module-based relationships were visualised using appropriate plots generated with SRPlot.

## 5. Conclusions

In conclusion, this comprehensive transcriptomic analysis establishes *O-*glycosylation as a key molecular determinant of TIME architecture in colorectal cancer, linking tumour-intrinsic glycosylation programmes to immune-desert phenotypes, genomic stability, MDR potential, and epithelial differentiation. These findings expand our understanding of the molecular underpinnings of immune heterogeneity in CRC and highlight *O-*glycosylation signatures as potential biomarkers for patient stratification and therapeutic targeting. Future studies integrating glycomics, spatial transcriptomics, and functional modelling will be essential to translate these observations into clinically actionable strategies for reprogramming the TIME.

## Figures and Tables

**Table 1:** Association of *O-*Glycosylation with tumour immune phenotypes.

Immune Phenotype	Standardised Test Statistic	*p*-Value	q-Value	Direction in High *O-*Glycosylation
Immune-inflamed	−6.268	<0.001	<0.001	Decreased
Immune-excluded	−2.697	0.007	0.02	Decreased
Immune-desert	2.816	0.005	0.016	Increased
Siglec Score	−3.94	<0.001	<0.001	Decreased

**Table 2:** Association of *O-*Glycosylation with MDR programmes.

MDR Programme	Standardised Test Statistic ⁱ	*p*-Value	Q-Value	Direction in High *O-*Glycosylation
MDR Efflux	+2.311	0.021	0.068	Increased
MDR Metabolic Inactivation	−2.730	0.006	0.019	Decreased
MDR Apoptosis Suppression	+1.230	0.219	0.337	Not significant
MDR Target Bypass Signalling	+8.287	<0.001	<0.001	Increased
MDR Stress Adaptation	+3.479	<0.001	0.002	Increased
MDR Xenobiotic Sensing	+6.707	<0.001	<0.001	Increased
MDR Trafficking/Sequestration	+9.485	<0.001	<0.001	Increased

ⁱ Standardised test statistic from the Mann–Whitney U test.

## Data Availability

All the data explored in this study are domiciled in the Genome Data Commons (https://portal.gdc.cancer.gov) and the cBioPortal for Cancer Genomics (https://www.cbioportal.org) databases (accessed 5 April 2026).

## Supplementary Materials

The following supporting information can be downloaded at: https://www.mdpi.com/article/10.3390/ph19060857/s1, Supplementary Materials S1: Table S1: Gene lists for generation of functional scores; Supplementary Materials S2: Table S2: Comparison of Four *O*-Glycosylation Cut-offs for Association with Clinical, Pathological, and Genomic Variables; Supplementary Materials S3: Table S3: Association of *O*-Glycosylation with Clinicopathological and Molecular Features in Colorectal Cancer; Supplementary Materials S4: Table S4: Association of *O*-Glycosylation with Siglec Scores and Tumour Phenotypic States; Supplementary Materials S5: Table S5: Sample Sizes (Total N) for Statistical Analyses of *O*-Glycosylation. Figure S1: Principal component analysis (PCA) plots before and after ComBat batch correction.
