# Supplementary material for: O-Glycosylation Signatures Shape the Tumour Immune Microenvironment and Associate with Genomic Stability, Drug Resistance Programmes, and Epithelial Differentiation in Colorectal Cancer
Source: Pharmaceuticals (Basel). 2026 May 29;19(6):857. doi: 10.3390/ph19060857 (PMC13304491; doi:10.3390/ph19060857)
Supplement: Supplementary file 1 [file pharmaceuticals-19-00857-s001.zip › Supplementary Figure S1.pdf]

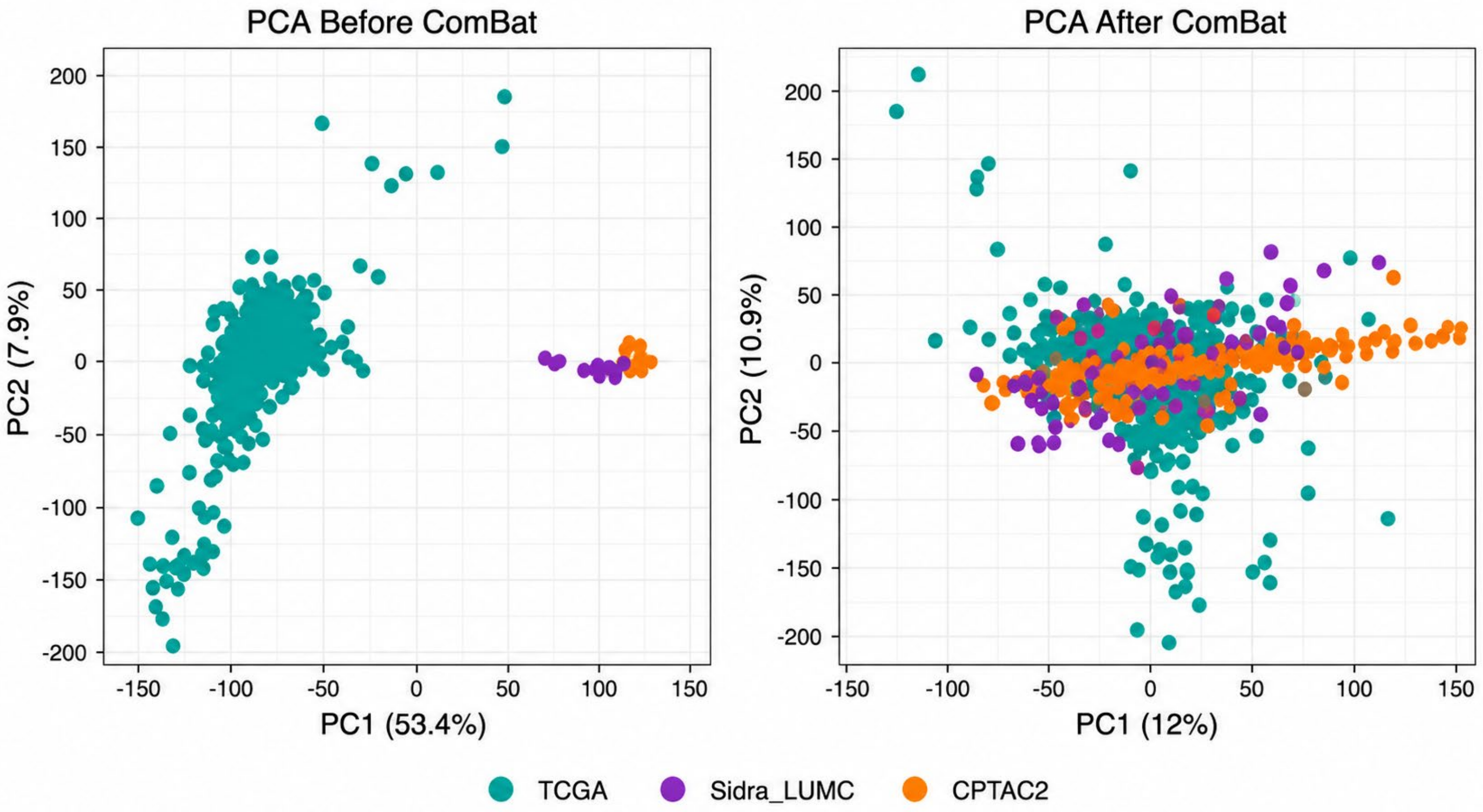

Supplementary Figure S1: **Principal component analysis (PCA) plots demonstrating the effect of ComBat batch correction across the integrated colorectal cancer transcriptomic cohorts (TCGA, CPTAC2, and Sidra-LUMC).** Left panel: PCA before batch correction showing pronounced cohort-specific clustering, indicating substantial batch effects between datasets. Before correction, PC1 explained 53.4% of the total variance and showed a strong correlation with batch assignment (PC1–batch correlation = -0.909648). Right panel: PCA after ComBat harmonization showing marked reduction in cohort-specific separation and improved overlap between samples from the three cohorts, consistent with effective correction of technical batch effects while preserving biological variation. After correction, PC1 explained 12.0% of the total variance and the PC1–batch correlation was substantially reduced (0.000625809). Each point represents an individual tumour sample coloured according to cohort origin.
